# Overview of the Genus *Boleodorus* and First Reports of *Boleodorus thylactus* and *B. volutus* from Southern Alberta, Canada

**DOI:** 10.3390/ani11061760

**Published:** 2021-06-12

**Authors:** Maria Munawar, Dmytro P. Yevtushenko, Pablo Castillo

**Affiliations:** 1 Department of Biological Sciences, University of Lethbridge, 4401 University Drive W, Lethbridge, AB T1K 3M4, Canada; maria.munawar@uleth.ca; 2 Institute for Sustainable Agriculture (IAS), Spanish National Research Council (CSIC), Campus de Excelencia Internacional Agrolimentario, ceiA3, Avenida Menendez Pidal s/n, 14004 Cordoba, Spain; p.castillo@csic.es

**Keywords:** associated hosts, ecological studies, morphology, morphometrics, nematode management programs, new record, soil health, plant parasitic nematodes, taxonomy

## Abstract

**Simple Summary:**

Two *Boleodorus* species were detected in cultivated areas of southern Alberta. The aim of the present work was to characterize the discovered populations of *Boleodorus* using morphological and molecular methods. *Boleodorus* is the least studied genus in family Tylenchidae, with very few species reported after formal descriptions and outside their type locality. To date, *Boleodorus* species are not considered nematode pest species, rather they can serve as environmental indicators. Therefore, it is important to quantify and monitor the population densities of these species for soil health management studies. The current study encompasses the distribution and host association of all described *Boleodorus* species. In addition, morphometrical characters of all valid species are listed for their prompt identification.

**Abstract:**

The present study provides the morphological and molecular characterization of *Boleodorus thylactus* and *B. volutus* populations, recovered from agricultural fields of southern Alberta. Despite a significant abundance of this group of nematodes, none of the *Boleodorus* species were previously reported in Canada. Therefore, representative adult specimens of each population were photographed and examined morphometrically. Phylogenetic trees were reconstructed using partial D2–D3 expansion segments of the 28S and 18S rDNA sequences to understand the relationships of *Boleodorus* species with Tylenchidae-related genera. *Boleodorus* species are relevant to soil ecological studies and therefore we summarized the important morphological and morphometric characters in tabular form for easy and efficient species identification. Moreover, we discuss the associated hosts and the distribution of all described *Boleodorus* species. This study will serve as a guide and basic framework for species diagnostics in the genus *Boleodorus* and will aid in filling the gaps in our knowledge of the species present in our cultivated lands.

## 1. Introduction

In southern Alberta, sustainable crop production is achieved not only through advanced agronomic practices but also through constant surveying and surveillance of the crop fields. The latter includes the collection of soil samples from cultivated areas and examination for the presence of plant-parasitic nematode species [1,2]. Various nematode species are associated with cultivated plants but only a select few of them are attributed importance in terms of crop yield reduction [3]. 

In addition to root-lesion nematodes, stunt and pin nematodes were recently reported in cultivated areas of southern Alberta [1,2,4]. During our recent survey, we isolated several populations of *Boleodorus* spp. that exhibited four lateral lines, curved to hooked tails, and delicate stylets. Since no *Boleodorus* species from Canada was previously reported, we elaborated on these findings by carrying out a detailed morphological and molecular analyses of these populations. By comparing morphometrical and morphological characters and molecular data we identified these species as *B. thylactus* Thorne [5] and *B. volutus* Lima and Siddiqi [6].

The genus *Boleodorus* was established by Thorne in 1941 [5] with the type species *B. thylactus*. Since then, several new members of this genus have been isolated and described from different crops and agricultural regions. Currently, this genus ranks as the second largest genus of subfamily Boleodorinae [7]. Although the members in this subfamily frequently occur in agricultural soils, they receive little attention compared with other plant-parasitic species [8]. The feeding habits of the majority of nematodes in this group are unknown, but members of the genus *Boleodorus* are considered epidermal cell or root hair feeders and are characterized either 1e or 2 on the colonizer-persister (cp) scale [9,10]. The nematodes in this category have short generations, high reproduction rates, and tolerance to ecological disturbances, thus serving as indicators of soil health [10]. *Boleodorus* species are also considered to be herbivores which rely on the roots of higher plants as their food source [11,12]. This highlights the fact that, even though *Boleodorus* species have short and delicate stylets, their feeding elicits a sort of mechanical injury to the roots. The genus *Boleodorus* is not recognized as being within the important soil pests that affect plants; however, having a non-parasitic status does not exclude *Boleodorus* species from disease management programs.

Literature studies indicated that *B. thylactus* was originally described in the USA, but also reported in several Asian and European countries [5,7,13]. The present study is the first report of *B. thylactus* and *B. volutus* found in the cultivated areas of southern Alberta, Canada. Despite its frequent occurrence and abundance, previous studies did not characterize the qualitative/quantitative characters and distribution pattern of *Boleodorus* species in a way that could stimulate more advanced research. Here, we carried out a detailed study of the morphological and morphometrical characters of the valid species of *Boleodorus*. In addition, we examined the phylogenetic relationships and distribution of the genus. The present study will serve as a guide and basic framework for species identification in the *Boleodorus* genus and will fill gaps in our knowledge of the species present in our cultivated areas.

## 2. Materials and Methods

### 2.1. Isolation and Morphological Studies

Soil samples were collected from different cultivated areas of southern Alberta. Nematodes were extracted from soil samples using the modified Cobb sieving and flotation-centrifugation method [14]. Mixed populations of *Boleodorus* species were detected in the samples from four different fields. *Boleodorus* members were collected individually from the mixture of soil nematodes and assigned the population numbers 40, 50, 61, and 62. For preliminary examinations, fresh *Boleodorus* adults were transferred to a drop of distilled water, heat relaxed at 60 °C and observed under a Zeiss Axioskope 40 microscope. Nematodes were fixed and permanent slides were prepared according to the methods of Seinhorst [15] and De Grisse [16]. Photo documentation of each specimen was carried out using a Zeiss Axioskope 40 microscope equipped with a Zeiss Axiocam 208 camera (Carl Zeiss Microscopy, Jena, Germany). Measurements on images were performed using ZEN blue 3.1 imaging software (Carl Zeiss Microscopy). 

### 2.2. DNA Extraction, PCR, and Sequencing

Nematode DNA was prepared according to Maria et al. [17]. Three sets of DNA primers (Integrated DNA Technologies, Coralville, IA, USA) were used in the PCR analyses to amplify the nucleotide sequences of the partial 18S, 28S (LSU), and ITS of ribosomal RNA genes (rDNA). The partial 18S rRNA region was amplified with 1813F and 2646R primers [18]. The LSU rDNA regions were amplified using 28–81F and 28–1006rev primers [19] and the ITS gene was amplified using F194 [20] and AB28-R primers [21]. The PCR conditions were as described in Holterman et al. [18,19] and in Ferris et al. [20]. Amplified PCR products were resolved by electrophoresis in 1% agarose gels and visualized by staining with GelRed (Biotium, Fremont, CA, USA). Amplified DNA fragments were purified using an E.Z.N.A. Gel Extraction Kit (Omega Biotek, Norcross, GA, USA) following the manufacturer’s instructions, ligated into the pJET1.2 vector (Thermo Fisher Scientific, Mississauga, ON, Canada), and introduced into *Escherichia coli* DH5α competent cells (Thermo Fisher Scientific). The presence of the PCR-derived inserts in the plasmids from transformed *E. coli* cells was confirmed by PCR. Plasmid DNA was isolated and purified using an E.Z.N.A. Plasmid DNA Mini Kit I (Omega Biotek), according to the manufacturer’s instructions, and sent to Genewiz, Inc. for DNA sequencing (South Plainfield, NJ, USA). DNA sequences were aligned using the BioEdit sequence alignment tool and compared for similarities with all known nematode species sequences in the GenBank database.

### 2.3. Phylogenetic Analyses

In the present study, D2–D3 expansion segments of 28S rRNA, 18S rRNA, and ITS rRNA sequences of the *Boleodorus* populations were obtained. These sequences and other sequences from species of Tylenchidae from GenBank were used for phylogenetic analysis. Selection of outgroup taxa for each dataset were based on previously published studies [22]. Multiple sequence alignments of the different genes were completed using the FFT-NS-2 algorithm of MAFFT v.7.450 [23]. The BioEdit program v7.2.5 [24] was used for sequence alignment visualization and edited using Gblocks v0.91b [25] on the Castresana Laboratory server (available online: http://molevol.cmima.csic.es/castresana/Gblocks_server.html (accessed on 21 April 2021)) using options for a less stringent selection (minimum number of sequences for a conserved or a flanking position: 50% of the number of sequences + 1; maximum number of contiguous non-conserved positions: 8; minimum length of a block: 5; allowed gap positions: with half). Phylogenetic analyses of the sequence datasets were based on Bayesian inference (BI) using MrBayes v3.1.2 [26]. The best-fit model of DNA evolution was achieved using JModelTest v2.1.7 [27] with the Akaike Information Criterion (AIC). The best-fit model, the base frequency, the proportion of invariable sites, and the gamma distribution shape parameters and substitution rates in the AIC were then used in MrBayes for the phylogenetic analyses. The transversion model with invariable sites and a gamma-shaped distribution (TVM  + I  + G) for the D2–D3 segments of the 28S rRNA and the transition model with a gamma-shaped distribution (TIM1 + G) for the 18S rRNA gene were run with four chains for 4 and 4 × 10^6^ generations, respectively. A combined analysis of the two ribosomal genes was not undertaken due to several sequences not being available for all species. The sampling for Markov chains was carried out at intervals of 100 generations. For each analysis, two runs were conducted. After discarding burn-in samples of 30% and evaluating convergence, the remaining samples were retained for more in-depth analyses. The topologies were used to generate a 50% majority-rule consensus tree. On each appropriate clade, posterior probabilities (PP) were given. FigTree software v1.42 [28] was used for visualization of trees from all analyses.

## 3. Results

### 3.1. Systematics

#### 3.1.1. Morphological and Morphometrical Observations on *Boleodorus* Species

This section briefly addresses the key diagnostic characters of *Boleodorus* species that can be used in species identification or discrimination. The majority of these species were described decades ago, with some original descriptions in languages other than English and often difficult to access. In this study, all original descriptions were collected through web search queries and personal communications with authors and journals’ editorial staff. By examining all the original descriptions, we found that important morphological and morphometrical characters—such as the presence/absence of males, the number of lateral lines, the presence/absence of crenations on the lip area or lateral field, lip and tail morphology, body and stylet length, and de Man ratios a, b, c, c′, and V—were discussed in almost all reports on *Boleodorus* species. Therefore, we summarize all these characters here and present them in Table 1 and Table 2; we anticipate that these efforts will aid in species identification of genus *Boleodorus*. Moreover, because the measurements presented in Table 1 and Table 2 were collected from the original descriptions, this information may help to identify intraspecific variations. Male body habitus and the morphology of the anterior and tail region were found to be similar to those of females; hence, only the important body ratios and measurements of spicule and stylet were included in Table 2.

In general, the females *of Boleodorus* occur much more abundantly than males [29], therefore priority was given to morphological characters and morphometrical values of females (Table 1). Apparently, males have not been described for nearly half of the *Boleodorus* species; very few reports acquired sufficient numbers of males for analysis. In most studies, fewer than 10 males were morphometrically characterized (Table 2). Interestingly, female spermathecae were reported to contain sperm in species described without males, which indicates that males are required for reproduction despite their notably lower numbers. The number of lateral lines has a diagnostic value in most nematode genera—all the described *Boleodorus* species have four lateral lines except *B. typicus* Hussain and Khan [30] and *B. zaini* Maqbool [31], both of which are reported to have six lateral lines. Moreover, the lateral field is generally smooth in this genus, however a few species (*B. constrictus* Rahman and Ahmad [32]*, B. filiformis* Hussain and Khan [33], *B. impar* Khan and Basir [34], and *B. mirus* Khan [35]) were reported to have crenations at the outer lines of the lateral field. 

The overall shape of the lip region in all the species ranges from conical to rounded and high to low, with or without depressions at the oral aperture. The lip region in *Boleodorus* species is smooth and without striations or annulation. Almost all the species are described as exhibiting a lip region continuous with the body contour, however some species—such as *B. cylindricus* Dhanachand, Renubala, and Anandi [36], *B. filiformis*, *B. innuptus* Andrassy [37], *B. modicus* Lal and Khan [38], *B. punici* Gambhir and Dhanachand [39], *B. solomonensis* Ye and Geraert [40], *B. spiralis* Egunjobi [41], *B. tenuis* Lal and Khan [38], *B. thylactus*, and *B. volutus*—are reported to have an offset lip region, though this offset is not by constriction or strong depression at the lip region.

The species of *Boleodorus* are not large worms; their body length ranges from 400 to 700 µm. The shortest species is *B. citri* Edward and Rai [42] (280–310 µm) and the longest is *B. minustylus* Mohilal, Anandi, and Dhanachand [43] (830–960 µm). The general body habitus is open C-shaped to spiral, however, *B. acurvus* Jairajpuri [44], *B. spinnocaudatus* Bina, Mohilal, Pramodini, and Majur-Shah [45], and *B. solomonensis* are outliers in this category and are reported to have almost straight body habitus. The stylets of *Boleodorus* species are delicate and short, ranging from 7 to 14 µm. The shortest stylet was reported for *B. minustylus* (4 µm) and the longest for *B. solomonensis* (12–14.5 µm). The excretory pore position is quite variable; however, it is always located in the region of the pharyngeal bulb or anterior to it. Only one species was exceptional, namely *B. zaini*, with the excretory pore located at the junction of the pharynx and intestine [31]. All the species of the genus have a monoprodelphic reproductive system and a post-uterine branch which is likely half a body width long. *Boleodorus* species can be regarded as dioecious—none are reported as hermaphroditic. 

The tail is the most variable character of this genus, with a length ranging from 46 to 101 µm, the longest of which was reported for *B. cylindricus* (88–101 µm). The general morphology of the tail is slender and ventrally curved. Only in some species (*B. azadkashmirensis* Maqbool, Shahina, and Firoza [46], *B. citri*, *B. constrictus*, *B. cynodeni* Fotedar and Mahajan [47], *B. innuptus*, *B. modicus, B. similis* Khan and Basir [48], *B. tenuis*, *B. thylactus*, and *B. volutus*) the terminal region of the tail is curved and hooked in shape. Three types of tail tips were described for *Boleodorus* species, i.e., rounded, pointed, and clavate. Almost all the species are reported to exhibit rounded tail tips except *B. citri*, *B. filiformis*, *B. flexuosus* Eroshenko [49], *B. longicaudatus* Bina, Mohilal, Pramodini, and Majur-Shah [45], and *B. spinnocaudatus*, which all have pointed tips, whereas *B. acurvus* and *B. clavicaudatus* Thorne [5] are known to have clavate tips.

The de Man ratios a, b, c, c′, and V are widely used and provided for all described species. The most significant ratio is the V-value, which can be used to differentiate species together with the other indices. Amphids, deirids, and phasmids were observed for very few species—in our opinion their absence or presence in a broader context does not seem to provide enough evidence for species separation or diagnostics.

Overall, *Boleodorus* species are small nematodes with short stylets, a valveless median bulb, a monodelphic gonad, small PUS, and slender, ventrally curved to hooked tails. There are not any morphological peculiarities to classify each *Boleodorus* species, therefore one should combine all morphological and morphometrical characters in order to distinguish species.

**Table 1:** Main morphological characters and morphometrics of female *Boleodorus* species. All measurements are in µm.

Species Name	Body Habitus	Lip Shape	Tail Shape	Tail Length	L	a	b	c	c′	V	Stylet Length	DGO	^2^ SE Pore Dist.	^3^ LL	References
1 *B. acurvus*	Almost straight	Continuous, narrow conical	Filiform, terminus clavate	75–90	470–540	27–37	4.2–4.7	6–9	7–9	60–63	10–11	4–6	80–90	4	Jairajpuri [44]
	Almost straight	Continuous, conical, depression at OA ^1^	Filiform, terminus rounded	77–93	475–605	27–39	4.1–5.1	5.7–6.9	7.2–8.5	62–64	10.5–11.5	–	79–95	4	Zeidan and Geraert [50]
2 *B. acutus*	Arcuate to open C-shape	Continuous, conical, elevated at perioral region	Uniformly conoid	63	500	22	–	8.0	–	67	13	–	–	4	Thorne and Malek [51]
3 *B. azadkashmirensis*	Open C-shape	Continuous, elevated, anteriorly truncated	Elongate-conoid, terminus hooked	60	400–500	23–33	4.0–5.0	7.0–8.0	4.6–5.8	63.5–67.6	10.4–12	1.5–2.0	67–80	4	Maqbool et al. [46]
4 *B. bambosus*	Arcuate	Slightly offset, raised, cupolate	Elongate-conoid, terminus rounded and curved	46–59	410–450	30–35	4.4–4.8	7.5–9.4	6.0–12	65.3–68.1	9.6–12.8	3.2	62.4–76.8	4	Mohilal et al. [43]
5 *B. citri*	Spiral	Continuous, cupolate, concave	Hooked, terminus subacute	46	280–310	21–23	–	7.3–12	–	64–68	9–10.5	–	55–61	4	Edward and Rai [42]
6 *B. clavicaudatus*	–	Continuous, conical	Conoid, terminus clavate	–	700	31	5.7	8.5	–	60	13	–	–	4	Thorne [5]
	–	Conical, anteriorly flattened	–	61–74	625–740	37–49	5.2–6.1	8.5–12	5.1–7.2	54.4–62.6	11–12.3	–	–	4	Sturhan and Hohberg [52]
7 *B. constrictus*	Spiral	Continuous, conical, depression at OA	Elongate-conoid, terminus hooked	68–85	490–550	32–37	4.6–5.4	6.4–8	6.4–9.0	63.3–67.2	11	2.5	–	4	Rahman and Ahmad [32]
8 *B. cylindricus*	–	Offset, elevated	Filiform, terminus rounded	88–101	480–610	37–47	–	6–7	9–13	63–64	6–8	7		4	Dhanachand et al. [36]
9 *B. cynodoni*	Close C-shape	Continuous, low, rounded	Elongate, terminus hooked	54	420–490	26–30	4.5–6.0	7–9	–	62–65	8	–	64	4	Fotedar and Mahajan [47]
10 *B. filiformis*	Arcuate	Slightly offset, cupolate, anteriorly flattened	Elongate, terminus acute	62	460–550	24–31	5.0–5.6	7–9	–	63–69	9	1.5	75	4	Husain and Khan [33]
11 *B. flexuosus*	Spiral	Slightly offset, high, not annulated	Elongate-conoid, curved, terminus pointed	91–96	550–570	25–28	–	5.7–6.1	–	61–65	10.5	4	–	4	Eroshenko [49]
12 *B. hyderi*	C-shape	Continuous, cupolate, flat	Elongate-conoid, terminus rounded	–	440–500	23–27	4.3–5.0	6.8–7.8	–	63.6–68.5	10–11	–	75–80	4	Husain and Khan [53]
13 *B. impar*	Close C-shape	Continuous, conoid, elevated	Elongate, ventrally arcuate	96	504–600	25–32	4.8–6.2	5–7	–	63–66	13–14	3	95	4	Khan and Basir [34]
14 *B. innuptus*	–	Offset, conical, not annulated	Ventrally arcuate, terminus hooked	59	470–490	25–29	–	7.3–8.3	–	64–66	11.5–12.5	–	–	4	Andrassy [37]
15 *B. longicaudatus*	Slightly ventrally arcuate	Continuous, conical, depression at OA	Elongate, terminus pointed	88–92	430–720	25–28	7.5–9.2	4.8–6.2	8.6–12	65–74	8.5	3.4	71–74	4	Bina et al. [45]
16 *B. minustylus*	Slightly curved ventrally	Continuous, rounded	Short, terminus rounded	59–72	960–830	48–52	5.6–7.4	10–14	5.3–7.5	79–81	4.0–4.8	3–5	99–109	4	Mohilal et al. [43]
17 *B. mirus*	Ventrally arcuate	Continuous, elevated	Elongate- filiform, terminus rounded	–	540–572	25–32	4.8–5.3	6.2–7.8	–	63–66	11–12	2	100	4	Khan [35]
18 *B. modicus*	Spiral	Slightly offset, raised, anteriorly-flattened, depression at OA	Hooked, terminus striated and rounded	51	429–487	24–31	4.2–5.0	7.7–9.8	4.0–5.5	66.5–71	9.8–10.2	3	77–78	4	Lal and Khan [38]
19 *B. neosimilis*	–	Continuous, narrow, truncate	Uniform, terminus blunt	51	460	23	–	9	–	68	10	–	–	4	Geraert [54]
20 *B. pakistanensis*	C-shape	Slightly offset, elevated, conoid, anteriorly truncated	Elongate-conoid, terminus rounded	73	540–580	31–34	5.0–5.2	7.5–8.0	–	67–68	11–12	4	–	4	Siddiqi [55]
21 *B. punici*	Slightly ventrally arcuate	Setoff, elevated, wide	Long, filiform, terminus rounded	73–82	450–510	37–43	4.9–6.6	5–7	9.6–12	58–64	7–8	7	–	4	Gambhir and Dhanachand [39]
22 *B. rafiqi*	C-shape	Continuous, cupolate, flat	Elongate, arcuate, terminus rounded	–	500–600	22–35	4.7–5.0	7.0–10	–	65–68	8–11	–	80–90	4	Husain and Khan [53]
23 *B. seshadrii*	Open C-shape	Continuous, conoid	Dorsally curved, terminus rounded	–	410–510	28–29	4.2–4.9	6.0–8.1	–	67–68	12–13	3	–	4	Handoo et al. [56]
24 *B. similis*	C-shape	Continuous, elevated	Elongate, terminus hooked	63	390–440	19–24	3.0–4.7	5–7	–	65–68	10–11	2	75	4	Khan and Basir [48]
25 *B. spinocaudatus*	Almost straight	Continuous, high and wide	Elongate, terminus rounded or pointed	49–80	600–690	25–36	6.6–11	7.5–13	4.6–6.7	76–83	8.5–10.2	5.1	62–88	4	Bina et al. [45]
26 *B. solomonensis*	Almost straight	Slightly offset, narrow, conical, depression at OA	Elongate, terminus rounded or clavate	80–93	550–650	24–39	4.5–6.0	6.3–7.3	6.1–9.3	53.4–58	12–14.5	–	85–98	4	Ye and Geraert [40]
27 *B. spiralis*	Spiral	Slightly offset, truncate	Short, terminus rounded	45	400–490	22–27	3.4–4.4	8.1–10	–	62.4–73.2	10–12	–	–	4	Egunjobi [41]
28 *B. thylactus*	Ventrally arcuate	Offset, convex conoid	Hooked, terminus acute	–	600	31	5.5	8.0		–	12.0	–	–	4	Thorne [5]
29 *B. tenuis*	Open C-shape	Slightly offset, raised, depression at OA	Hooked, terminus unstriated and rounded	71	480–550	25–30	4.6–5.2	6.3–7.6	6.0–7.7	65–70	11.3–12.5	2.5	75–84.6	4	Lal and Khan [38]
30 *B. teres*	Ventrally arcuate	Continuous, conoid-truncate	Ventrally curved, terminus rounded	71	420–560	23–35	–	6.0–8.5	6.5–10	54–63	10–12	5	90	4	Nanjappa and Khan [57]
31 *B. typicus*	Open C-shape	Continuous, truncated, cupolate	Elongate-conoid, terminus rounded	–	440–580	23–27	3.9–5.3	6–9	–	62–68	12–14	–	90–100	6	Husain and Khan [30]
32 *B. volutus*	Spiral	Offset, raised, cupolate, anteriorly flattened	Hooked, terminus unstriated and rounded	35–60	450–510	20–28	4.9–5.7	8.7–12	–	67–72	8.3–9.2	–	78	4	Lima and Siddiqi [6]
33 *B. zaini*	Spiral	Continuous, high, smooth, truncated	Conoid, terminus rounded	87	640–670	27–30	5.7–7.2	7.6–9.5	6.5–7.3	65.2–67.3	12.6	4	–	6	Maqbool [31]

^1^ Oral aperture; ^2^ Distance from anterior end to excretory pore; ^3^ Number of lateral lines in lateral field.

**Table 2:** Main morphological characters and morphometrics of male *Boleodorus* species. All measurements are in µm.

	Species Name	*n*	L	a	b	c	Stylet Length	Spicule Length	Spicule Morphology	Gubernaculum	Bursa	References
1	*B. acurvus*	1	460	38	4.2	5	–	16	Cephalated, arcuate	–	Adanal	Jairajpuri [44]
		1	520	37	4.4	6.1	10.5	12.5	–	5.0	Adanal	Zeidan and Geraert [50]
2	*B. azadkashmirensis*	10	400–440	25–44	4.0–4.6	6.6–7.8	10.4–11.2	12.8–16	Cephalated, arcuate	4.0–4.8	Crenated, adanal	Maqbool, Shahina, and Firoza [46]
3	*B. clavicaudatus*	1	635	39	5.6	9.6	11.5	14	–	–	–	Sturhan and Hohberg [52]
4	*B. cylindricus*	–	–	–	–	–	–	16	–	3–6	–	Dhanachand, Renubala, and Anandi [36]
5	*B. cynodoni*	4	390–450	32–35	4.5–6.0	6–7	8	10–11	–	4	Crenate, short	Fotedar and Mahajan [47]
6	*B. filiformis*	1	460	38	4	6	9	13	Paired, cephalated, arcuate	3	Crenate, adanal	Husain and Khan [33]
7	*B. flexuosus*	–	–	–	–	–	–	14	–	–	–	Eroshenko [49]
8	*B. hyderi*	1	380	22	4.1	7.0	10	12	–	5	–	Husain and Khan [53]
9	*B. impar*	6	520–560	37–47	4–6	5–6	12–14	18–20	Paired, cephalated, arcuate	7–8	Adanal	Khan and Basir [34]
10	*B. minustylus*	2	660–670	52–53	5.3–5.8	11–15	3–4	14–16	Sclerotized and arcuate	4.8	–	Mohilal, Anandi, and Dhanachand [43]
11	*B. mirus*	5	525–590	37–47	5–6	4–4.8	10–11	19–22	Paired, cephalated, arcuate	9–10	Crenated	Khan [35]
12	*B. neosimilis*	–	–	–	–	–	–	–	Cephalated	14	–	Geraert [54]
13	*B. punici*	2	470–500	34–35	5.2–5.8	5.0–5.7	7–9	13–16	–	2–3	–	Gambhir and Dhanachand [39]
14	*B. similis*	1	380	26	4.2	5	11	15	Cephalated, arcuate	3	Crenated	Khan and Basir [48]
15	*B. solomonensis*	6	505–590	25.5–35.0	4.2–5.4	5.7–6.6	12–13	14–17	Paired, cephalated	–	Adanal	Ye and Geraert [40]
16	*B. thylactus*	1	500	33	5.0	7.2	–	–	–	–	–	Thorne [5]
		14	535–662	30.2–42.3	4.8–6.3	8.2–11.5	10.9–12.4	8.8–11.1	–	–	–	Deimi and Mitkowski [58]
17	*B. tenuis*	10	440–530	25.0–31.6	4.5–5.0	6.5–6.9	10.8–12.0	12–14.2	Arcuate	5.0	–	Lal and Khan [38]
18	*B. teres*	–	–	–	–	–	–	13	Cephalated	6	Crenate	Nanjappa and Khan [57]
19	*B. typicus*	5	520–560	33–47	4.5–5.3	7	13–14	18–20	Paired, cephalated	3–5	Adanal	Husain and Khan [30]
20	*B. volutus*	1	440	28	4.9	9.6	9.0	14–15	Cephalated, arcuate	4.8–5.1	Adanal	Lima and Siddiqi [6]
21	*B. zaini*	9	480–600	30–33	5.3–6.2	7.5–9	12.6	16–18	Paired, arcuate	6.7	Short, adanal	Maqbool [31]

#### 3.1.2. Description of *B. thylactus* Thorne, 1941

*Female*: Body habitus ventrally arcuate to C-shaped after heat relaxed. Lateral field with four equidistant lines, inner lines not as faint as described in the majority of other species. Lip region slightly offset, conical rounded in shape, anteriorly flattened with depressions at oral aperture. Stylet short, delicate with flange-like knobs, pharyngeal dorsal gland orifice (DGO) close to the stylet knob base. Pharynx composed of long and narrow corpus, metacorpus (i.e., median bulb) indistinct, valveless, irregularly shaped. Isthmus narrow, encircled with nerve ring ending in pyriform basal pharyngeal bulb. Excretory pore located in the pharyngeal bulb region, hemizonid indistinct, located 2–3 body annuli anterior to excretory pore, visible in fixed specimens. Cardia hemispherical. Intestine thick, appears filled with granules. Gonad monoprodelphic, vulva a transverse slit, vagina extending into uterine sac, crustaformeria indistinct, columnar arrangements of cells not clearly distinguishable, spermatheca offset, filled with sperm, oviduct composed of large cells, ovary short and composed of multiple rows. Post-vulval uterine sac small, composed of undifferentiated cells, half of the maximum body diameter long. Anus distinct, appears as oblique line. Tail region slender, posterior half of the tail curved ventrally to form hooked shape, tail tip rounded in the majority of specimens, pointed tip also observed in a few.

*Male*: Body habitus and anterior region similar to females. Spicule arcuate to curved, 16–19 µm long. Tail morphology similar to that of females. Bursa adanal, starting at the head of spicule and ending one spicule length posterior to cloaca. Phasmids not found in any of the specimens. 

*Juveniles*: Juveniles are present in each studied sample. Only a few juveniles were handpicked and observed under the light microscope. They are similar to adults in general appearance except for under-developed pharyngeal and reproductive components. Since immature forms do not have enough characters to enable diagnosis, they were therefore excluded from the morphometrical analyses.

**Figure 1:**
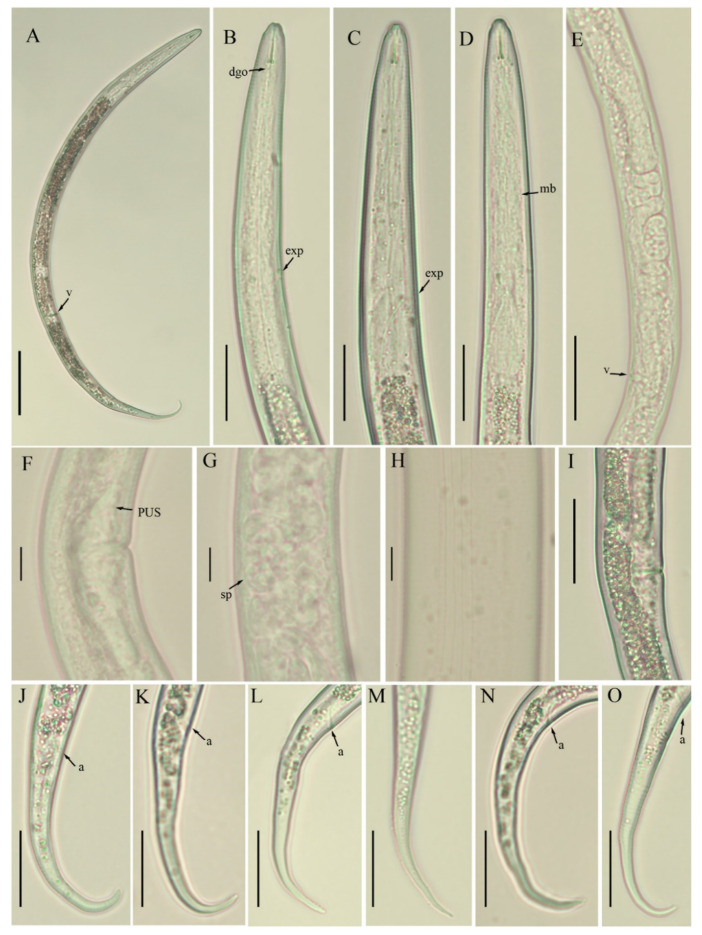
Photomicrographs of *B. thylactus* female, Canadian population 61. (**A**) Entire body; (**B**–**D**) pharyngeal region; (**E**) gonad; (**F**) vulval region; (**G**) spermatheca; (**H**) lateral lines; (**I**) vulval region; (**J**–**O**) tail regions. Scale bars: (**A**) 50 µm; (**B**–**E**,**I**–**O**) 20 µm; (**F**–**H**) 5 µm. Arrowheads: (a) anus; (exp) excretory pore; (dgo) dorsal esophageal gland orifice; (mb) median bulb; (sp) spermatheca; (PUS) post uterine sac; (v) vulva.

**Figure 2:**
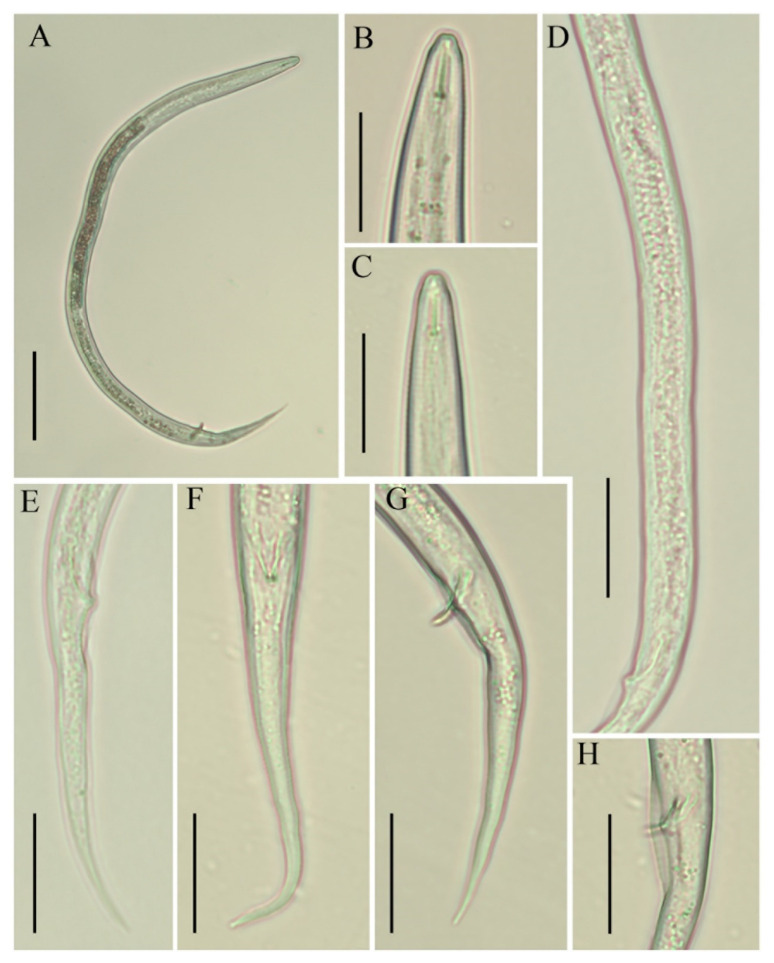
Photomicrographs of *B. thylactus* male, Canadian population 61. (**A**) Entire body; (**B**,**C**) lip region; (**D**) gonad; (**E**–**G**) tails; (**H**) cloacal region with bursa. Scale bars: (**A**) 50 µm; (**B**–**H**) 20 µm.

**Figure 3:**
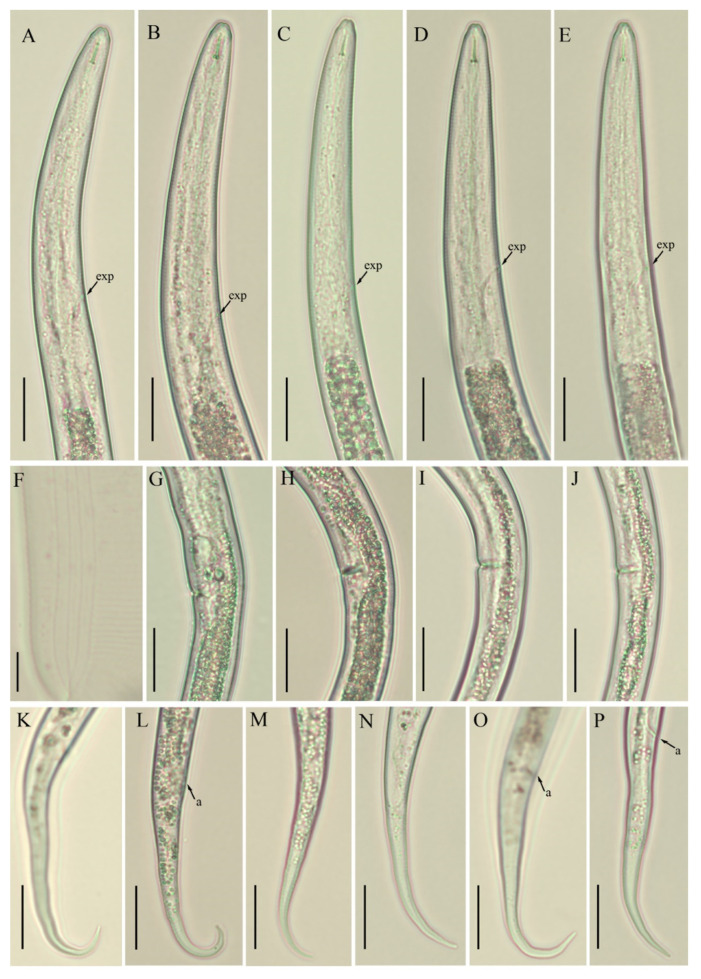
Photomicrographs of *B. thylactus* female, Canadian populations 40, 50, and 62. (**A**,**B**; **C**; **D**,**E**) pharyngeal region of population 40; 50; and 62, respectively; (**F**) lateral lines; (**G**; **H**,**I**; **J**) vulval regions of populations 40; 50; and 62, respectively; (**K**,**L**; **M**,**N**; **O**,**P**) tail regions of populations 40; 50; and 62, respectively. Scale bars: (**A**–**E**, **G**–**P**) 20 µm; (**F**) 5 µm. Arrowheads: (a) anus; (exp) excretory pore.

*Remarks*: Though *B. thylactus* is reported in different countries, morphometrics were provided for only a few populations (Table 1, Table 2 and Table 3, Figure 1, Figure 2 and Figure 3). 

The morphology and morphometry of Alberta populations of *B. thylactus* agree well with the original and other reports, except for the Brazilian population which is the smallest amongst all the reported populations (Table 3). The presence of males was reported in the original and Belgian populations, but a formal characterization was not provided in any of the reports. The population from Iran supplied the detailed morphometrics of males but no morphological characterization was provided by the authors [58]. In our study, males were present and were characterized both morphologically and morphometrically; however, males were not abundant in each studied sample. Considering this, we speculate that males have little diagnostic importance in this species. *B. thylactus* was originally described in the USA and was later reported from Afghanistan, Belgium, Brazil, Germany, India, Iran, Slovakia, and Spain in the rhizosphere of cultivated plants, grasses, and fruit trees. This species was also reported from meadows and arable lands, which indicates its ability to survive in any soil type and vegetation (Table 3). In the present study, we recovered four populations of *B. thylactus* from the cultivated areas of southern Alberta, making ours the first report of *B. thylactus* from Canada.

**Table 3:** Morphometrics of *B. thylactus* from Canada and other countries. All measurements are in µm and presented as mean ± standard deviation (range).

	Canada (Present Study)					Thorne [5]	Geraert [59]	Rashid et al. [60]	Lal and Khan [38]	Deimi and Mitkowski [58]	
Populations	61		40	50	62	USA ^1^	Belgium	Brazil	India	Iran	
Characters	Females	Males	Females	Females	Females	Female	Females ^2^	Female	Females ^3^	Females	Male
*n*	15	5	10	10	10	–	–	1	–	12	14
Body length	525.3 ± 31.5 (483–602.0)	477.3 ± 23.7 (450.0–500.0)	501.3 ± 32.7 (446–552)	527.1 ± 28.9 (490.0–576.0)	507.5 ± 34.3 (450.0–558.0)	600	380–590	390	430–580	598 (535–662)	544 (501–614)
a	32.5 ± 2.3 (28.5–35.9)	38.5 ± 1.9 (36.9–41.2)	33.0 ± 22.1 (37.2–31.5)	31.8 ± 2.5 (27.3–34.4)	32.8 ± 2.8 (27.8–37.9)	31	21–39	25	23–29	35.4 (30.2–42.3)	41.3 (37.6–47.2)
b	4.8 ± 0.3 (4.3–5.5)	4.4 ± 0.2 (4.2–4.6)	4.7 ± 7.5 (4.5–4.9)	4.9 ± 0.3 (4.6–5.5)	4.8 ± 0.3 (4.3–5.3)	5.5	4–6.4	4.4	4.8–6.2	5.2 (4.8–6.3)	5.6 (4.6–5.8)
c	9.0 ± 1.1 (7.0–10.2)	6.8 ± 0.2 (6.6–7.0)	8.1 ± 5.1 (8.1–7.7)	8.7 ± 1.1 (7.0–10.1)	8.0 ± 0.9 (6.6–9.1)	8	5.6–8.7	7.4	6.7–8.2	9.7 (8.2–11.5)	8.8 (7.2–9.4)
c′	5.9 ± 0.8 (5.0–8.0)	7.9 ± 0.7 (6.9–8.4)	6.4 ± 9.2 (6.1–6.7)	6.2 ± 0.8 (5.2–7.7)	6.5 ± 1.0 (5.0–8.1)	–	–	6.4	5.9–7.8	6.4 (4.6–7.9)	7.4 (6.4–9.1)
V	67.0 ± 1.3 (65.0–69.0)	31.2 ± 1.7 (29.2–32.4)	66.2 ± 1.1 (65.0–68.0)	66.8 ± 1.5 (65.0–69.0)	66.5 ± 1.4 (65.0–69.0)	–	62–71.5	65	64–68	64.1 (61.8–65.9)	–
MB	41.9 ± 4.2 (37.5–48.6)	56	–	–	–	–	–	–	–	–	–
G1	25.7 ± 2.2 (23.1–30.6)	–	22.0 ± 24.8 (22.2–21.2)	22.4 ± 1.9 (21.1–23.8)	21.5 ± 1.7 (19.5–22.6)	–	–	–	–	–	–
Lip height	2.8 ± 0.2 (2.4–3.0)	3.0 ± 0.1 (3.0–3.1)	2.9 ± 0.2 (2.6–3.3)	2.7 ± 0.2 (2.4–2.9)	3.0 ± 0.2 (2.5–3.3)	–	–	–	–	–	–
Lip width	5.5 ± 0.3 (5.1–6.0)	5.3 ± 0.3 (5.0–5.7)	5.9 ± 0.2 (5.4–6.0)	5.4 ± 0.3 (5.1–5.9)	5.6 ± 0.3 (5.0–5.9)	–	–	–	–	–	–
Stylet length	9.3 ± 0.8 (8.2–10.7)	9.0 ± 1.2 (7.5–10.3)	9.4 ± 0.5 (8.5–10.6)	9.6 ± 0.8 (8.6–10.8)	9.6 ± 0.3 (9.2–10.0)	12	8.5–12	10	10.5–12	10.9 (10.1–12.3)	11.1 (10.9–12.4)
Anterior end to excretory pore	84.8 ± 5.1 (74.0–92.0)	82.3 ± 3.9 (77.0–86.0)	85.1 ± 4.5 (79.0–92.0)	88.3 ± 4.2 (85.0–96.0)	82.2 ± 3.1 (79.0–87.0)	–	–	–	–	82.6 (80.7–87.8)	80.2 (78.7–84.1)
Pharynx length	109.1 ± 3.9 (103.0–117.0)	109.5 ± 1.7 (108.0–111.0)	107.3 ± 4.4 (99.0–113.0)	107.2 ± 6.0 (99.0–117.0)	105.0 ± 5.1 (99.0–113.0)	–	–	88.5	–	108 (100–119)	101 (99–107)
Maximum body width	16.3 ± 1.9 (13.8–20.0)	12.4 ± 0.6 (12.0–13.3)	15.2 ± 1.5 (12.0–17.5)	16.6 ± 1.2 (15.0–18.0)	15.6 ± 1.4 (13.5–18.0)	–	–	16	–	15.7 (13.4–17.5)	12.6 (12.1–14.3)
Vulva body width	14.8 ± 1.6 (13.0–18.5)	–	14.4 ± 1.3 (12.0–17.0)	15.3 ± 1.1 (14.0–17.0)	14.4 ± 1.1 (13.0–16.0)	–	–	–	–	–	–
Post uterine sac length	11.1 ± 0.9 (9.7–13.0)	–	10.2 ± 1.1 (9.0–12.0)	9.7 ± 1.1 (8.2–11.6)	9.4 ± 0.8 (8.2–10.8)	–	–	–	–	10.7 (8.5–14.6)	–
Distance from vulva to anus	111.6 ± 9.0 (102.0–131.0)	–	106.9 ± 4.2 (101.0–115.0)	112.7 ± 4.1 (106.0–118.0)	104.8 ± 6.3 (101.0–119.0)	–	–	–	–	107.8 (98–119)	–
Anal/cloacal body width	10.2 ± 1.4 (8.8–13.0)	8.9 ± 1.0 (8.1–10.3)	9.7 ± 0.7 (9.0–10.8)	10.0 ± 0.9 (8.1–10.9)	9.8 ± 0.8 (9.0–11.0)	–	–	–	–	–	–
Spicule length	–	17.0 ± 1.4 (16.0–19.0)	–	–	–	–	–	–	–	–	9.5 (8.8–11.1)
Tail length	59.4 ± 9.5 (50.0–77.0)	70.3 ± 1.7 (68.0–72.0)	61.9 ± 6.4 (55.0–72.0)	61.5 ± 6.3 (52.0–71.0)	63.9 ± 7.7 (55.0–75.0)	–	–	53	–	67 (59–82)	68.6 (66.8–70.7)

^1^ Original description; ^2^ Composite values of two populations consisting of 15 and 9 females; ^3^ Composite values of two populations consisting of 20 and 10 females.

#### 3.1.3. Description of *B. volutus* Lima and Siddiqi, 1963

*Female*: Body habitus ventrally arcuate to close C-shaped after heat relaxed. Lateral field with four equidistant lines. Lip region elevated, slightly offset, conical rounded in shape, anteriorly flattened with slight depressions at oral aperture. Stylet short, delicate with flange-like knobs, DGO close to the stylet knob base. Pharynx composed of long and narrow corpus, metacorpus (i.e., median bulb) indistinct, valveless, irregularly shaped. Isthmus narrow, encircled with nerve ring ending in pyriform basal pharyngeal bulb. Excretory pore located in the region of pharyngeal bulb, hemizonid indistinct, located 2–3 body annuli anterior to excretory pore. Intestine thick, appears filled with granules. Gonad monoprodelphic, vulva a transverse slit, vagina extending into uterine sac, crustaformeria indistinct, spermatheca offset. Post uterine sac small, composed of undifferentiated cells, half of the maximum body diameter long. Anus distinct, appears as oblique line. Tail ventrally curved, hook-shaped ending in rounded tip. 

*Male and juveniles*: Not found.

**Figure 4:**
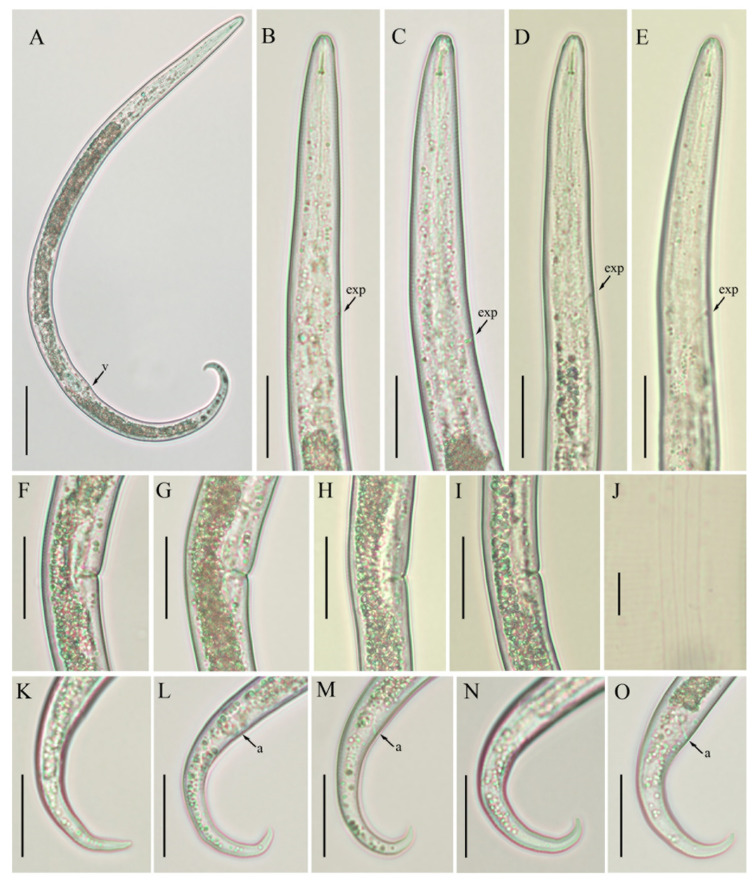
Photomicrographs of *B. volutus* female, Canadian populations 61 and 40. (**A**) Entire body, population 61; (**B**,**C**; **D**,**E**) pharyngeal region of populations 61 and 40, respectively; (**F**,**G**; **H**,**I**) vulval regions of populations 61 and 40, respectively; (**J**) lateral lines; (**K**–**M**; **N**,**O**) tail regions of populations 61 and 40, respectively. Scale bars: (**A**) 50 µm; (**B**–**I**,**K**–**O**) 20 µm; (**J**) 5 µm. Arrowheads: (a) anus; (exp) excretory pore; (v) vulva.

*Remarks*: *B. volutus* was originally described from England in the rhizosphere of grass [6]. Subsequently, it was reported from the Netherlands [61] and Poland [62]. Unfortunately, both references are inaccessible, so it is not possible to compare the morphometrics or additional details associated with these reports. Recently, *B. volutus* was reported from Afghanistan [63] and Germany [52] in the rhizosphere of cultivated crops and grasslands, but without any photo documentation or morphometric data. The scarcity of morphometric and image data explains the difficulties in dealing with *Boleodorus* species. In the present study, we found four populations or *B. volutus* in cultivated areas of southern Alberta; however, we only studied the morphology of two populations. The other two populations did not contain sufficient adult individuals to carry out morpho-molecular studies. Morphometrically, the Canadian population of *B. volutus* is slightly longer and wider than the original description (Table 4). The rest of the characters, such as lip morphology, stylet length, and tail shape, agree well with the original description. *B. volutus* is morphologically very similar to *B. thylactus*, however, both species can be differentiated from each other by lip and tail morphology. In addition, our sequence analyses indicate that both species are molecularly distant (Figure 4).

### 3.2. Habitat and Locality

Both populations of *B. thylactus* and *B. volutus* were present in all four fields. The geographical locations of each field are as follows. Population 40: latitude 49°46′3.684″ N; longitude −112°24′13.3056″ W; Municipal District of Taber, Alberta, Canada. Population 50: latitude 49°47′22.7256″ N; longitude −111°59′50.082″ W; Municipal District of Taber, Alberta, Canada. Population 61: latitude 49°50′52.4868″ N; longitude −111°22′34.0752″ W; Rural Municipality of Forty Mile County, Alberta, Canada. Population 62: latitude 49°57′24.6276″ N; longitude −111°18′24.5556″ W; Rural Municipality of Forty Mile County, Alberta, Canada. Fields 50 and 61 were covered with grasses, whereas fields 40 and 62 had green manure cover crop and canola, respectively. The soil type of fields ranged from sandy to clayey. Regardless of the soil type, *B. thylactus* was the dominant species compared with *B. volutus*.

### 3.3. Distribution and Associated Hosts of Boleodorus Species

*Boleodorus* species appear to have a diverse host range and geographical distribution. The majority of species of this genus were originally described from India (20 spp.), the USA (4 spp.), Pakistan (3 spp.), Hungary, the Solomon Islands, New Zealand, Nigeria, Russia, and the UK (Table 5; [7]). Once formally described, only *B. acurvus*, *B. clavicaudatus*, *B. pakistanensis* Siddiqi [55], *B. thylactus*, and *B. volutus* were reported outside of their type locality [3,12,50,52,58]. 

There are some additional reports in which species level identification was not carried out and only generic presence was reported [64,65]. Species level resolution is imperative, as each species has a different ecological role and properties [10,66]. The associated hosts of *Boleodorus* species range from agronomic/horticultural crops to grasses, perennial plants, meadows, and arable lands. Based on these reports, we speculate that the diversity of *Boleodorus* species has not been fully explored; if more attention is given to this genus, together with other principle parasitic species, there will likely be more reports of *Boleodorus* species.

**Table 4:** Morphometrics of *B. volutus* female. All measurements are in µm and presented as mean ± standard deviation (range).

Character	Canadian Populations		Original Description
Populations	61	40	Lima and Siddiqi [6]
*n*	10	8	16
Body length	547.4 ± 31.5 (501.0–581.0)	533.0 ± 30.7 (487.0–577.0)	480 (450–510)
a	27.4 ± 2.5 (24.5–31.3)	28.9 ± 2.6 (24.9–32.1)	26 (20–28)
b	4.6 ± 0.2 (4.2–4.8)	4.5 ± 0.2 (4.2–4.9)	5.3 (4.9–5.7)
c	9.6 ± 0.8 (8.5–10.6)	10.0 ± 1.0 (8.8–11.5)	10 (8.7–12)
c′	5.4 ± 0.8 (4.3–6.3)	5.2 ± 0.5 (4.6–5.8)	–
V	68.9 ± 0.7 (68.0–70.0)	69.0 ± 1.2 (68.0–71.0)	69 (67–72)
Lip height	2.7 ± 0.2 (2.4–3.0)	2.3 ± 0.3 (2.1–2.8)	–
Lip width	5.9 ± 0.2 (5.4–6.3)	5.6 ± 0.1 (5.5–5.8)	–
Stylet length	8.5 ± 0.4 (8.0–9.3)	9.2 ± 0.6 (8.2–9.8)	8.7 (8.3–9.2)
Anterior end to excretory pore	90.7 ± 4.2 (86.0–97.0)	88.4 ± 3.8 (85.0–95.0)	78
Pharynx	118.5 ± 4.7 (109.0–125.0)	119.0 ± 7.3 (108.0–129.0)	–
Maximum body width	20.1 ± 2.4 (16.0–22.7)	18.6 ± 2.3 (16.0–22.4)	–
Vulva body width	17.4 ± 1.7 (15.0–19.8)	16.9 ± 1.4 (15.0–19.0)	–
Post uterine sac length	10.4 ± 1.7 (7.2–12.6)	9.6 ± 1.2 (8.0–12.1)	–
Distance from vulva to anus	118.1 ± 8.1 (105.0–129.0)	110.5 ± 7.3 (102.0–121.0)	–
Anal body width	10.9 ± 1.3 (9.5–13.2)	10.3 ± 0.7 (9.2–11.0)	–
Tail length	57.8 ± 3.0 (52.0–62.0)	53.9 ± 4.8 (46.0–59.0)	–

**Table 5:** Distribution and associated hosts of *Boleodorus* species.

	Species Name	Country	Host	References
1	*B. acurvus*	Nigeria	*Saccharum officinarum* L.	Jairajpuri [44]
		Sudan	*Citrus limon*	Zeidan and Geraert [50]
2	*B. acutus*	USA	–	Thorne and Malek [51]
3	*B. azadkashmirensis*	Pakistan	*Allium cepa*, *Zea mays* *Fragaria ananassa*	Maqbool, Shahina, and Firoza [46]
4	*B. bambosus*	India	*Bambusa tuida*	Mohilal, Anandi, and Dhanachand [43]
5	*B. citri*	India	*Citrus reticulata*	Edward and Rai [42]
6	*B. clavicaudatus*	USA	Alfalfa crowns	Thorne [5]
		German	Loamy soil from meadows	Sturhan and Hohberg [52]
7	*B. constrictus*	India	*Carica papaya*	Rahman and Ahmad [32]
8	*B. cylindricus*	India	*Saccharum officinarum* L.	Dhanachand, Renubala, and Anandi [36]
		India	*Brassica oleracea*	Hassan and Ahangar [67]
9	*B. cynodoni*	India	*Cynodon dactylon* Pers.	Fotedar and Mahajan [47]
10	*B. filiformis*	India	*Solanum melongena*	Husain and Khan [33]
11	*B. flexuosus*	Russia	–	Eroshenko [49]
12	*B. hyderi*	India	*Mangifera indica* L.	Husain and Khan [53]
13	*B. impar*	India	*Cynodon dactylon* Pers.	Khan and Basir [34]
14	*B. innuptus*	Hungary	–	Andrassy [37]
15	*B. longicaudatus*	India	*Morus alba* L.	Bina, Mohilal, Pramodini, and Majur-Shah [45]
16	*B. minustylus*	India	Banana roots	Mohilal, Anandi, and Dhanachand [43]
17	*B. mirus*	India	*Cynodon dactylon* Pers.	Khan [35]
18	*B. modicus*	India	*Populus* sp. and *Myrica sapida*	Lal and Khan [38]
19	*B. neosimilis*	USA	-	Geraert [54]
20	*B. pakistanensis*	Pakistan	*Pinus excelsa* Wall.	Siddiqi [55]
		Egypt	*Polypogon monspeliensis* L., *Solanum nigrum* L.,	Ibrahim et al. [3]
21	*B. punici*	India	*Punica granatum*	Gambhir and Dhanachand [39]
22	*B. rafiqi*	India	*Pyrus communis* L.	Husain and Khan [53]
23	*B. seshadrii*	India	*Glycine max* L.	Handoo et al. [56]
24	*B. similis*	India	*Plumeria acutifolia* Poir.	Khan and Basir [48]
25	*B. spinocaudatus*	India	*Morus alba* L.	Bina, Mohilal, Pramodini, and Majur-Shah [45]
26	*B. solomonensis*	Solomon Islands	Moist soil in tropical forest, host unknown	Ye and Geraert [40]
27	*B. spiralis*	New Zealand	Dominant vegetation composed of *Leptospermum scoparium*, *Weinmannia racemose,* and *Psuedopanax arboreum*	Egunjobi [41]
28	*B. thylactus*	USA	Cultivated soil, alfalfa crown roots	Thorne [5]
		Belgium	Meadows, arable land	Geraert [59]
		Brazil	*Theobroma cacao*	Rashid et al. [60]
		India	*Pinus* sp., *Artocarpus integrifolia*	Lal and Khan [38]
		Spain	Natural plant communities	Castillo et al. [13]
		Iran	Grapes	Karegar et al. [68] Deimi and Mitkowski [58]
		Afghanistan	Clover	Asghari et al. [63]
		Germany	Arable soil, cherry tree plantation	Sturhan and Hohberg [52]
		Iran	*Polianthes tuberosa*	Husseinvand et al. [69]
		Slovakia	*Solidago gigantea*	Čerevková et al. [12]
29	*B. tenuis*	India	*Casurina equsitifolia*	Lal and Khan [38]
30	*B. teres*	India	–	Nanjappa and Khan [57]
31	*B. typicus*	India	*Narcissus* sp.	Husain and Khan [30]
32	*B. volutus*	UK	Grass species	Lima and Siddiqi [6]
		Netherlands	–	Bongers [61]
		Poland	–	Brzeski [62]
		Afghanistan	Potato, tomato	Asghari et al. [63]
		Germany	Meadow soil	Sturhan and Hohberg [52]
33	*B. zaini*	Pakistan	*Citrus aurantium*	Maqbool [31]

### 3.4. Molecular Characterization of B. thylactus and B. volutus with Phylogenetic Relationships of Boleodorus with Related Genera

Both *Boleodorus* species were sequenced for partial 18S (MZ081056–MZ081059), D2–D3 of 28S (MZ081091–MZ081097), and ITS (MZ099822–MZ099823) regions. Thirteen new sequences were obtained in the present study.

**Figure 5:**
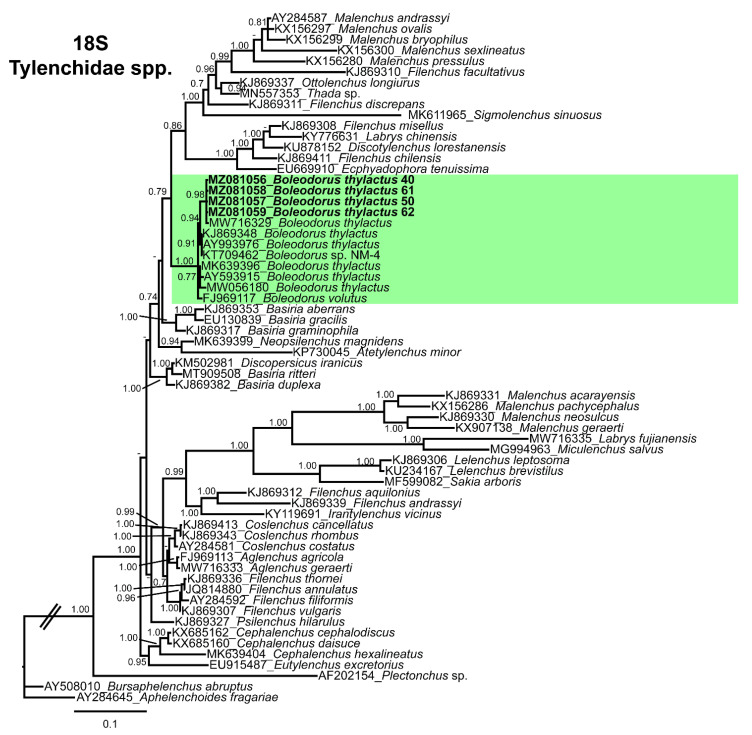
Phylogenetic relationships of the genus *Boleodorus* spp. within Tylenchidae. Bayesian 50% majority rule consensus tree as inferred from 18S rRNA gene sequence alignment under the transition model with a gamma-shaped distribution (TIM1 + G). Posterior probabilities of more than 0.70 are given for appropriate clades. Sequences newly obtained in this study are shown in bold. The scale bar indicates expected changes per site. *Boleodorus* spp. clade in green color.

The partial 18S sequences of Canadian *B. thylactus* (MZ081056–MZ081059) showed 98–99% (1–11 bp and 0–1 indels difference,) sequence identity with the *B. thylactus* (MW716329, KJ869348, AY993976, KT709462, MK639396, AY593915, MW056180) sequences deposited in NCBI (Figure 5). In addition to that, Canadian *B. thylactus* showed 99% (4 bp difference and 0 indels) sequence identity with the *B. volutus* (FJ969117) from the Netherlands.

**Figure 6:**
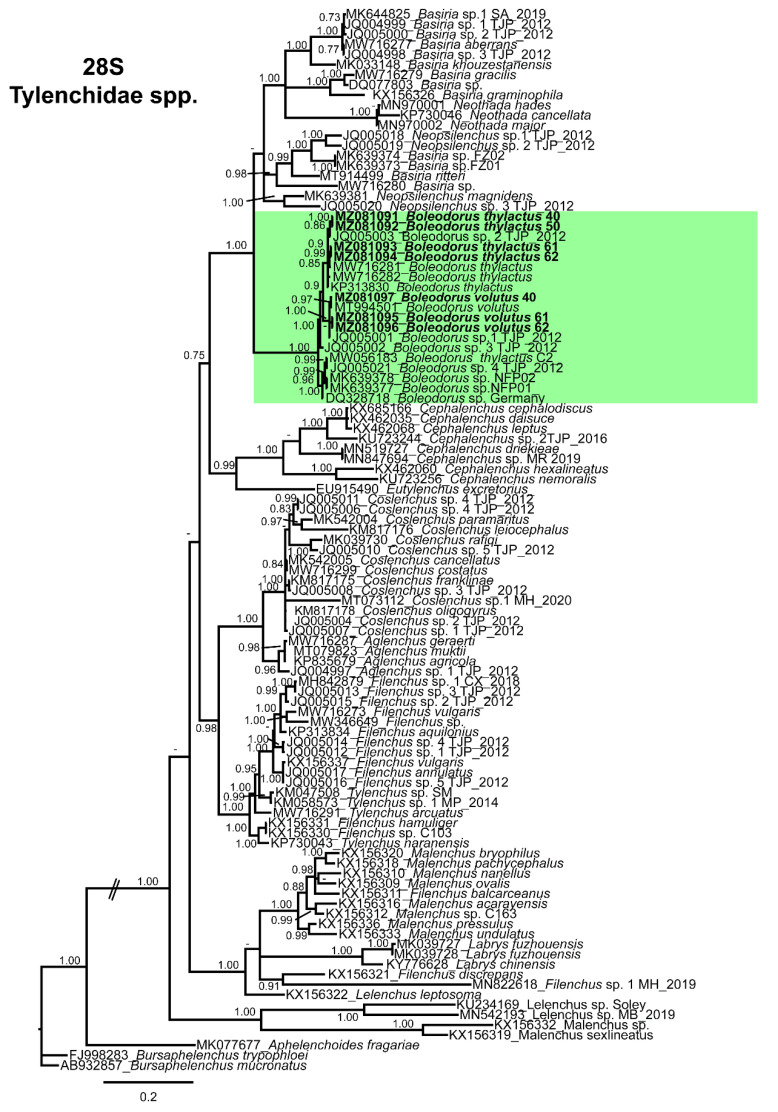
Phylogenetic relationships of the genus *Boleodorus* spp. within Tylenchidae. Bayesian 50% majority rule consensus tree as inferred from D2–D3 expansion domains of the 28S rRNA sequence alignment under the transversion model with invariable sites and a gamma-shaped distribution (TVM  + I  + G). Posterior probabilities of more than 0.70 are given for appropriate clades. Sequences newly obtained in this study are shown in bold. The scale bar indicates expected changes per site. *Boleodorus* spp. clade in green color.

The D2–D3 of 28S (MZ081091–MZ081094) obtained for Canadian *B. thylactus* showed 95–98% (8–37 bp and 0–1 indels difference) sequence identity with the *B. thylactus* (MW716281, MW716282, KP313830, MW056183) sequences deposited in NCBI (Figure 6). Moreover, Canadian *B. thylactus* showed 94–99% (10–39 bp and 0–1 indels difference) sequence similarity with unidentified *Boleodorus* spp. (JQ005001–JQ005003, JQ005021, MK639378, MK639377, DQ328718) and 96% (28 and 0 indels difference) sequence similarity with *B. volutus* (MT994501) from the USA. The D2–D3 of 28S (MZ081095–MZ081097) sequences obtained for Canadian populations of *B. volutus* showed 99% (8 bp and 0 indels difference,) sequence identity with the *B. volutus* (MT994501) from the USA.

Phylogenetic analyses were performed for only two markers (18S and D2–D3 of 28S). ITS sequences were obtained for *B. thylactus*, however, due to lack of similarity with other Tylenchidae genera, phylogenetic analysis cannot be performed for this marker. The nucleotide BLAST results of ITS sequences showed 84–87% similarity with *Ditylenchus* sp. (MF669512, MF669513 from Taiwan), *Filenchus* sp. (MH842880 from China), and *Coslenchus rhombus* (MK874505 from South Africa), with very low sequence coverage of 26–56% and poor E-value.

The 18S tree was performed with 64 sequences of Tylenchidae species (four of them new, belonging to *B. thylactus*) and three outgroup taxa *Plectonchus* sp. (AF202154), *Bursaphelenchus abruptus* Giblin-Davis, Mundo-Ocampo, Baldwin, Norden, and Batra [70] (AY508010), and *Aphelenchoides fragariae* (Ritzema Bos) Christie [71,72] (AY284645). The Bayesian 50% majority rule consensus tree inferred from the partial 18S alignment is given in Figure 5. The tree contained a highly supported major clade (PP = 1.0) comprising all the species of the genus *Boleodorus*, including the *B. thylactus* and *B. volutus* from Alberta, Canada (Figure 5). The D2–D3 domains of the 28S rRNA gene alignment (699 bp long) included 98 sequences of Tylenchidae species (four of them new, belonging to *B. thylactus*, and three others from *B. volutus*) and three outgroup species, *Bursaphelenchus trypophloei* Tomalak [73] (FJ998283), *Bursaphelenchus mucronatus* Mamiya and Enda [74] (AB932857), and *Aphelenchoides fragariae* (MK077677). The Bayesian 50% majority rule consensus tree inferred from the D2–D3 alignment shows one highly supported major clade (PP = 1.0) comprising species of *Boleodorus*, including *B. thylactus* and *B. volutus* from Alberta, Canada (Figure 6). In both our phylogenetic analyses, none of the *Boleodorus* species held doubtful positions; both our species grouped within the *Boleodorus* clade. From these results, it seems that *Boleodorus* is a monophyletic genus, but we also consider the possibility that its phylogenetic positioning could change after more *Boleodorus* species sequences become available for phylogenetic study.

## 4. Discussion

The present study focuses on morphology/morphometry and phylogenetics of the least studied tylenchid nematode, namely, *Boloeodorus* species. Several studies have been published on different Tylenchidae genera, such as *Basiria* [75,76], *Malenchus* [22,77] *Filenchus* [78], and *Cephalenchus* [79], to improve our knowledge and understanding of this complex group of nematodes. However, no detailed studies were carried out for *Boleodorus*.

In general, *Boleodorus* species plays an essential role in soil ecological studies. Moreover, these species were detected in the rhizosphere of agricultural and horticultural plants [9,10,58,65]. The association of *Boleodorus* with plants has not been clearly demonstrated or assessed except by Geraert [59], who reported 150 individuals of *B. thylactus* in 100 mL of soil collected from arable lands of Belgium, and Deimi and Mitkowski [58], who reported the same species in a vineyard of Iran with a frequency as high as 80%. Since then, such a high density of nematodes has not been observed in subsequent reports; *B. thylactus* and *B. volutus* were found in clover and potato fields of Afghanistan at a frequency of 36 and 12%, respectively [63]. *Boleodorus pakistanensis* was frequently found present in the rhizosphere of grass and flowering plants in Egypt [3].

*Boleodorus cylindricus* was detected in cauliflower with a 90% frequency and infestation rate of 36% [67]. Similarly, *Boleodorus* was found to be the most abundant genus present in commercial vegetable fields of Srinagar, India [64]. Additionally, Pan et al. [65] conducted studies on the nematode communities in the black soil region of China, reporting that *Boleodorus* was the most abundant genus in both grass and bare lands. Moreover, Čerevková et al. [12] carried out a study in Slovakia to assess the effect of an invasive plant (*Solidago gigantean* Aiton) on the soil nematode communities. They found that *B. thylactus* and some other nematodes were abundantly present in the invasive sites as compared with noninvasive ones, suggesting that vegetation type partly shapes nematode communities. In our study, we found mixed populations of *B. thylactus* and *B. volutus* in the cultivated areas of southern Alberta. We detected adults and juveniles of *B. thylactus* in each sample with a density of 15–40 individuals/100g of soil. Despite the presence of a *B. volutus* in each sample, the number of individuals was very low. Because composite samples were prepared from a mixture of 30 core samples collected at a depth of 30 cm within each field, it is likely that areas with a high density of *B. volutus* either were missed or diluted significantly with other soils. Other than *Boleodorus*, we also detected root-lesion, spiral, and pin nematodes; the identification of other detected nematodes will be part of our future studies.

While analyzing published data on *Boleodorus* species, we found that some *Boleodorus* species were inadequately described, requiring synonymization or further detailed studies to retain valid taxa status. In our work, we do not propose any taxonomical revisions, rather we list all the important characters that one should consider while performing *Boleodorus* identification. Moreover, we agree with several nematologists [2,52,80] that such actions should only be performed after recollection of type material and conducting a detailed molecular study using ribosomal and mitochondrial markers [81]. Like other Tylenchidae nematodes, the current sequence-based data for *Boleodorus* species are insufficient. Very few *Boleodorus* sequences are present in NCBI for comparative and phylogenetic studies; most of the sequences consist of unidentified *Boleodorus* species. In our phylogenetic analyses, none of the *Boleodorus* species held a doubtful position, consequently allowing us to conclude that *Boleodorus* species may be monophyletic. However, it is prudent to consider that only a small portion of *Boleodorus* species have been discovered or are available for sequence-based study; the phylogenetic positioning will likely change in the future.

## 5. Conclusions

Surprisingly, no *Boleodorus* species were previously reported from Alberta, Canada. The latest reports on nematode studies focus on those groups of nematodes that are considered pests of economic relevance [1,2,4]. In this study, we presented morphological data and distribution of a nematode genus which, so far, does not hold pest status. Knowledge of nematode species present (or absent) in a plant growing area is important to the growers, because each cropping system hosts different nematode species and may require different soil and crop management strategies. Moreover, the results of our study will aid researchers to correctly identify species that were previously unknown or escaped detection in prior field surveillance programs.

## Data Availability

Not applicable.
